# 239. Comparing Alternative Treatment Options for *Stenotrophomonas maltophilia* bacteremia

**DOI:** 10.1093/ofid/ofad500.312

**Published:** 2023-11-27

**Authors:** Nicholas S Teran, Andrei Zidaru, Kady Phe

**Affiliations:** Baylor St. Luke's Medical Center & University of Houston, Houston, Texas; Baylor St. Luke's Medical Center, Houston, Texas; Baylor St. Luke's Medical Center, Houston, Texas

## Abstract

**Background:**

*Stenotrophomonas maltophilia* can cause nosocomial infections in immunocompromised hosts and patients with indwelling devices or broad-spectrum antibiotic exposure. Treatment of *S maltophilia* infections poses a challenge due to intrinsic resistance mechanisms, including inducible L1 metallo and L2 cephalosporinase beta-lactamases. Trimethoprim-sulfamethoxazole (SXT) is recognized as the agent of choice for *S maltophilia* infections. Outcomes with alternative antimicrobials are not well described.

**Methods:**

We conducted a retrospective study of adults with positive blood cultures with *S maltophilia* who received ≥48h of directed antimicrobials at our institution between 3/2013 and 6/2022. Patients were stratified by their antimicrobial treatment, including levofloxacin, SXT, minocycline, ceftazidime, or combination therapy. The primary outcome was 30d all-cause mortality. Secondary outcomes were microbiological and clinical cure. Microbiological failure was defined as blood cultures growing *S maltophilia* during or within 7d of discontinuing therapy. Clinical failure was defined as a lack of resolution of signs or symptoms of infection requiring therapy adjustment.

**Results:**

Of 67 patients with positive *S maltophilia* blood cultures, 53 patients treated with levofloxacin (n = 27), SXT (n = 10), minocycline (n = 8), combination (n = 5) or ceftazidime (n = 3) were included. The remaining 14 patients were excluded for receiving < 48h of therapy. The median Charlson comorbidity index was 4 [IQR 3-6]. The most common source of bacteremia was catheter-related (n = 27). Overall, 30d mortality was 26.4%. There was no significant difference in 30d mortality among patients treated with levofloxacin, SXT, minocycline, combination or ceftazidime (p = 0.23). Interestingly, patients with monomicrobial *S maltophilia* bacteremia had statistically higher 30d mortality (35.3% versus 11.8%, p < 0.05). Microbiological and clinical cure rates were similar between treatment groups (p = 0.06 and p = 0.16, respectively).

**Conclusion:**

Treatment options for *S maltophilia* bacteremia resulted in similar 30d mortality; however, findings were limited by the small sample size. Larger, prospective studies are warranted to detect differences in treatment alternatives.

**Disclosures:**

**All Authors**: No reported disclosures

